# Effectiveness of Water-Based Exercise in Patients with Chronic Obstructive Pulmonary Disease: Systematic Review and Meta-Analysis

**DOI:** 10.3390/s23208557

**Published:** 2023-10-18

**Authors:** María Jesús Benzo-Iglesias, Patricia Rocamora-Pérez, María Ángeles Valverde-Martínez, Amelia Victoria García-Luengo, Remedios López-Liria

**Affiliations:** 1 Health Research Centre, Department of Nursing, Physiotherapy and Medicine, University of Almería, Carretera del Sacramento s/n, La Cañada de San Urbano, 04120 Almería, Spainmvm637@ual.es (M.Á.V.-M.); 2 Random Models and Design of Experiments, Department of Mathematics, University of Almería, Carretera del Sacramento s/n, La Cañada de San Urbano, 04120 Almería, Spain

**Keywords:** aquatic exercise, chronic obstructive pulmonary disease (COPD), functional capacity, lung capacity, physiotherapy

## Abstract

Chronic obstructive pulmonary disease (COPD) is a progressive respiratory disease that, due to dyspnea, decreases patients’ physical function and quality of life. The aim of the research was to evaluate the effectiveness of water-based exercise (WE) in improving functional capacity and respiratory muscle strength in patients with COPD. It consisted of a systematic review and meta-analysis of eight randomized clinical trials (RCTs) from the last 10 years, found in PubMed, PEDro, Scopus and Web of Science databases. Methodological quality was analyzed using the PEDro scale and the Cochrane Collaboration Risk of Bias Tool. Regarding the evaluation of functional capacity, mainly assessed were lung function, respiratory muscle strength, and maximal or aerobic exercise. The results showed that WE improves functional capacity compared to a non-exercising control group (SMD: 73.42; IC 95%: 40.40 to 106.45; I^2^: 0%). There are no statistically significant differences between a WE treatment and a land exercise (LE) treatment (*p* = 0.24) in functional capacity, nor with respect to respiratory muscle strength (*p* = 0.97). These data should be interpreted with caution, as more RCTs with aquatic intervention in COPD patients are needed to elucidate whether there are differences between WE or LE according to patient characteristics and comorbidities.

## 1. Introduction

Chronic obstructive pulmonary disease (COPD) is a common, preventable and treatable, but not fully reversible, chronic progressive respiratory disease. It is characterized by airflow restriction caused by abnormalities in the airways and/or pulmonary alveoli, which is usually generated by significant exposure to noxious particles or gases [1,2,3,4,5]. People with COPD have shortness of breath, cough, and recurrent exacerbations, and frequently suffer dyspnea both at rest and during exertion, which decreases their physical functioning and quality of life, affecting them with long-term comorbidities [1,2,3,4,5].

COPD is the name of a group of lung diseases, including emphysema and chronic bronchitis. According to the World Health Organization (WHO), it occurs in adults from the age of 35 [5,6,7], and its prevalence in people over the age of 40 has reached 13.7%. Nearly three billion people worldwide are at risk of COPD, and it is estimated that there will be more than five million deaths per year and related diseases by 2060 [2].

COPD is a major cause of morbidity, mortality and healthcare costs worldwide [3,4,5], being the third leading cause of death in 2019 according to WHO, and its prevalence is expected to increase due to population aging [8,9].

The diagnosis is considered in people with risk factors, who routinely experience symptoms such as coughing, wheezing, dyspnea and frequent chest infections, and is corroborated by spirometry in which a forced expiratory volume in the first second (FEV_1_)/forced vital capacity (FVC) ratio after bronchodilator of less than 70% shows persistent airflow limitation/obstruction [5,6,7,8]. 

There is evidence to affirm that COPD patients have systemic damage which is secondary to primary pulmonary involvement, which manifests itself in peripheral muscle involvement and a deficit of postural control, compared to healthy subjects of the same age [5,7,10].

Traditional COPD treatments focus on symptom control and exacerbation management. However, recently, pulmonary rehabilitation protocols have aimed at a more functional and comprehensive approach, emphasizing increased participation, improved exercise tolerance, enhanced quality of life and reduced hospitalizations to cut healthcare costs and mortality. The initial symptom management typically combines lifestyle changes, pharmacological treatment and respiratory physiotherapy [4,5,7,8]. 

Land exercise training (LE) has proven effective in enhancing functional capacity, quality of life and reducing hospital admissions and stays for COPD patients [3,11,12,13,14]. Yet not all COPD patients can engage in LE due to age-related and physical comorbidities [3].

Water exercise (WE) is recognized for its therapeutic potential in preventing and treating various conditions, capitalizing on the properties of water [15]. Water may be the most suitable medium for exercise in people with musculoskeletal or orthopedic comorbidities [2,3].

Aerobic WE, thanks to the loading effect on respiratory muscles, may also benefit lung function, dyspnea and respiratory muscle strength in COPD patients [2,4,16,17]. However, the meta-analysis by McNamara (2013) [3] suggests that there may be no significant differences between LE or WE in terms of exercise capacity or pulmonary function improvement [2,3]. 

While previous research has explored aquatic therapy in COPD patients, it often focused on specific exercises (aerobic exercises) [2] or aspects of body function (balance) [5], and only a limited number of studies were reviewed, which may lead to less robust and more limited conclusions compared to reviews that include a larger number of studies [3]. The present review aims to consolidate scientific evidence from the last decade regarding the effectiveness of any type of WE on physical and pulmonary function in COPD patients.

The objective of this systematic review was to evaluate the effectiveness of WE in improving functional capacity and respiratory muscle strength in patients with COPD.

## 2. Materials and Methods

### 2.1. Design

A systematic review was carried out taking into account the Preferred Reporting Items for Systematic Reviews (PRISMA) [18]. This review has been registered in the PROSPERO international prospective registry of systematic reviews (CRD42023394407).

### 2.2. Data Sources and Searches

To answer the proposed objectives, an electronic search was performed in January 2023, in PubMed, PEDro, Scopus and Web of Science databases, with no restrictions on language or publication state. The filter “publication date” of 10 years (2012 to 2023) was used in PubMed, Scopus and Web of Science databases. Three simultaneous searches were carried out in each database with the following descriptors: “aquatic therapy”, “chronic obstructive pulmonary disease”, “aquatic exercise” and “water based exercise”, joined by the Boolean operator “AND”. MESH terms (Medical Subject Headings) were not considered during the search in any of the databases. The search strategy relied solely on keywords and text-based queries to identify relevant articles. While MESH terms can be valuable for precision, in this case, the decision was made to use a keyword-based approach to maintain simplicity and minimize the risk of missing potentially relevant studies. 

### 2.3. Selection Criteria

To select the articles, the following PICOS eligibility criteria [19] were used:

- Participants: persons with COPD confirmed via diagnosis with Global Initiative for Obstructive Lung Disease (GOLD) criteria (FEV1/FVC < 70%), without age restriction.
- Intervention: studies were included with any type of exercise or physical activity in water (e.g., strength exercise, aerobic exercise or a specific therapy method), with no restrictions on dose, frequency or duration of treatment.
- Comparison: control groups with other interventions or no intervention, such as WE versus LE, WE versus usual medical therapy, or WE versus no intervention.
- Outcome: physical and pulmonary function measurements such as 6 min walk test (6MWT), incremental walk test (ISWT), FEV1 via spirometry, up and go test (UGT), respiratory muscle strength via maximal inspiratory and expiratory pressure, muscle strength via dynamometer, among others.
- Study Design: randomized clinical trials (RCTs). The selection of RCTs is due to the fact that systematics reviews and meta-analyses that are based on RCTs provide the highest level of evidence for healthcare decision making [20].

Inclusion criteria: RCTs between 2012 and 2023 (last 10 years), in any language. Limiting the systematic review to RCTs from the last 10 years ensures up-to-date and relevant evidence for current clinical practice, reflecting methodological improvements and clinical changes. This approach aims to capture studies conducted with improved methodologies and which address contemporary healthcare questions. Studies in which patients made use of medications (co-intervention) were accepted.

Exclusion criteria: Quasi-experimental clinical trials, pilot studies, preliminary studies or study protocols and systematic reviews with or without meta-analysis were not considered. Also excluded were studies that did not address COPD and WE, or those that were not related to the objectives of this review.

Search strategies and article selection are presented in Table 1.

### 2.4. Study Selection, Data Extraction and Risk of Bias Assessment

Two independent authors (MJBI and PRP) made the selection of references by examining the full text according to the inclusion and exclusion criteria. A third researcher (RLL) was consulted if there was any disagreement. The author (MJBI), using a standardized table, subtracted the characteristics of each study: number of participants, mean age, percentage of men and women, data from the WE intervention group (experimental group, EG), comparison intervention (CG), characteristics of the aquatic environment, duration of treatment, outcome measures or variables, and results obtained from the study.

The PEDro scale [29] was used to assess the methodological quality of the RCTs, and the Cochrane Collaboration Risk of Bias Tool [30] was used to assess the risk of bias.

### 2.5. Meta-Analysis

Meta-analyses of the main measured variables (6MWT, maximal inspiratory pressure (MIP), maximal expiratory pressure (MEP)) were performed using Review Manager software (RevMan version 5.4.1), including three to five of the eight RCTs according to variable. The I^2^ statistic was used to determine the degree of heterogeneity: 25% = low, 50% = medium and 75% = high heterogeneity. If I^2^ was 50%, a random-effects model would be used; and the mean difference was calculated for a 95% confidence interval. Several forest plots were generated to illustrate the overall effect of the interventions on each measure.

## 3. Results

**Figure 1:**
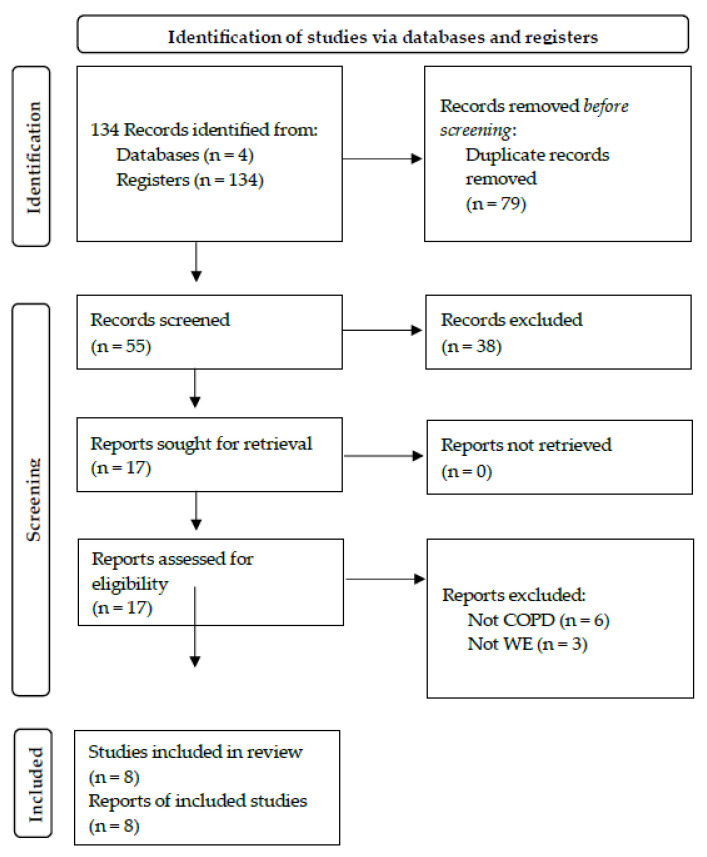
Flow diagram for article selection. RCT: randomized controlled trial; COPD: chronic obstructive pulmonary disease.

The different database searches offered 134 records. After eliminating duplicate articles, 55 records were analyzed, of which 47 were excluded following the inclusion and exclusion criteria. Finally, 8 articles were selected according to the objective of the study (Figure 1). 

Eight RCTs published in English were included, with a total of 298 participants. Table 2 shows the characteristics of each RCT selected, including information on the participants, interventions, aquatic medium, duration, variables measured and outcomes obtained.

### 3.1. Participants

Age ranged from 54 to 82 years, except for the study by De Souto Araujo [22], in which age only reached up ranged up to 49 years. The mean age of the studies was 67 years.

With regard to sex, the total percentage of men (in the sample of 298) was 61.74%, and the percentage of women was 38.26%. In practically all the studies, there was a greater proportion of men to women, except in two studies where the opposite was true [21,27]. In addition, in the Gallo-Silva study [28], only men were included.

Four of the studies included smokers [21,23,26,27]; De Souto Araujo [22] did not include smokers, and the rest did not report this characteristic [24,25,28].

In six of the studies included, participants did not have serious comorbidities that could affect physical exercise [22,23,24,25,26,27], whereas in McNamara (2015) [21] and McNamara (2013) [27], they did present physical comorbidities. 

### 3.2. Type of Intervention

The groups who performed LE engaged in aerobic exercise [21,22,23,26,28], strength exercise [22,23,26] or Liuzijue exercise [24,25] as the main part of the training. The intensity at which the exercises were performed, according to the modified Borg scale for dyspnea and perceived exertion, was 3–5 for two of the studies [21,27] and 4–6 in three studies [24,25,26,28].

Liuzijue is a traditional Chinese therapeutic exercise that consists of performing different slow movements of the upper limbs (UL) and lower limbs (LL) during exhalation to produce six different sounds (xu, he, hu, si, chui and xi), which are easy to learn and perform, with the advantage that there are no limitations due to the environment or equipment [24,25]. Abdominal-diaphragmatic breathing and pursed-lip exhalation are characteristic of Liuzijue and can increase the depth of breathing and respiratory time (of inspiration and expiration) and decrease the respiratory rate for the improvement of abnormal breathing patterns that occur in people with COPD. In addition, the movements can increase exercise tolerance and decrease the sensation of dyspnea [24,25].

Of the eight studies included in the review, five performed aerobic exercise [21,22,23,26,28], three performed strength exercise [22,23,26] and two performed Liuzijue exercise [24,25]. In addition, a warm-up was performed beforehand and a cool-down afterwards that both included breathing exercises and stretching. The level of water immersion was reported in most studies: in four, it was at the level of the xiphoid appendix and clavicle [21,24,25,27], and in two, at the level of the umbilicus [23,26]. The physiotherapist/patient ratio was indicated in four studies [21,23,27,28]. 

### 3.3. Outcomes Measures

Three of the eight studies have assessed lung function using FEV_1_, FVC and Tiffeneau index (FEV_1_/FVC) [22,23,25], although all studies performed lung function assessment to record baseline characteristics of participants. Five studies assessed respiratory muscle strength using the MIP and MEP [22,23,25,26,27]. Three studies assessed peripheral musculature strength: Felcar [23], via 1RM in UL (biceps and triceps) and LL (quadriceps); Wu study [25], via isokinetic dynamometer in UL (biceps and triceps) and LL (quadriceps and hamstrings); and De Castro [26], via isometric dynamometer in LL (quadriceps).

Functional capacity was assessed via the 6MWT in six of eight studies [22,23,24,26,27,28] and the 30 s stand up test (30″SST) in Liu’s study [24]. Maximal exercise capacity was assessed in four studies [23,24,26,27] via the ISWT [23,26,27] and in Liu [24] via a cycloergometer. Aerobic exercise capacity was assessed in McNamara (2013) [27] via the aerobic endurance walking test (ESWT). De Castro [26] assessed both static and dynamic balance, using a force platform and the UGT, respectively.

Several studies assessed mortality through the BODE Index [22,23], which records body mass index (BMI), exercise capacity, degree of airway obstruction and dyspnea to predict survival of COPD patients. Health-related quality of life (HRQOL) was assessed with the modified St George’s Hospital Respiratory Disease Questionnaire (SGRQ) [22,24,28] and the Chronic Respiratory Disease Questionnaire (CRDQ) [23,27]. In addition, two studies assessed anxiety and depressive symptoms through the Hospital Anxiety and Depression Questionnaire (HADQ) [23,27]. 

The McNamara (2015) study [21] recorded participants’ preferences for aquatic or land-based exercise. Five studies collected the presence or absence of adverse effects [21,24,25,26,27].

### 3.4. Intervention Effects

#### 3.4.1. Water Exercise (WE) versus Land Exercise (LE)

Upon assessing functional capacity with the 6MWT or the 30″SST, all studies found a significant improvement in both WE and LE groups [22,23,24,26,27]. De Souto Araujo [22] indicated that there was a decrease in the rate of perceived exertion for the LE group and a decrease in the dyspnea index for the experimental group (EG). 

Four studies assessed maximal exercise capacity [23,24,26,28]. Two studies found a significant improvement in the EG and the LE [23,26], but in the study by Felcar [23], the improvement in the EG occurred at 3 months, while the LE improvement occurred at 6 months. The study by McNamara (2013) [27] obtained a significant improvement in the EG compared to the LE. Liu [24] found that the EG improved volume of oxygen (VO_2_), and both the EG and the LE improved maximal work rate.

Four studies showed a significant improvement in the strength of the respiratory muscle force (MIP and MEP) both in the EG and in the LE [22,23,25,26], although the study by Felcar [23] found that the improvement of the MIP occurred at 3 months in the EG and at 6 months in LE; De Castro [26] specifies that the improvement of the MEP was greater in the EG than in the LE. In both groups, McNamara (2013) [27] showed no significant differences for either MIP or MEP.

Regarding pulmonary function [22,23,25], two studies found no significant differences [23,25], but De Souto Araujo [22] found an improvement in FEV1 in both intervention groups.

McNamara (2013) [27] assessed aerobic exercise capacity, showing that the EG obtained a significant improvement compared to the LE.

Felcar [23] assessed peripheral muscle strength via 1RM, finding a significant improvement in EG and LE at 3 months of intervention. Wu [25] assessed this measure via isokinetic dynamometer and showed that in ULs, both the EG and the LE improved total flexor and extensor work; and in LLs, the EG and the LE significantly increased peak torque, peak torque/body weight and total extensor work, in addition to increasing total flexor work in the EG. De Castro [26] assessed strength using an isometric dynamometer, obtaining significant improvement in both the EG and the LE.

Two studies evaluated HRQOL using the SGRQ [22,24] and showed a significant improvement in all domains for the LE. Although not significant, De Souto Araujo [22] found an improvement for the EG, whereas Liu [24] did not find a significant improvement for this group. Two other studies evaluated HRQOL using the CRDQ [23,27]. In one, Felcar [23] shows a significant improvement of both groups at 3 months, while McNamara (2013) [27] only finds significant differences in the fatigue domain, in favor of the EG compared to the LE.

Two studies assessed the BODE mortality predictor index [22,23], where Souto Araujo [22] highlighted a decrease in both intervention groups (and an increase in the CG without exercise), while Felcar [23] showed no significant changes.

Anxiety and depression symptoms using the HADQ [23,27] did not show significant changes in the intervention groups.

Regarding static balance, no significant changes were found in any of the groups, but there was a significant improvement in the EG in dynamic balance assessed via UGT [26].

In three studies, no adverse effects were found in any of the intervention groups [24,25,26]. However, in two studies, adverse effects were reported [21,27]: in the EG, they consisted of generalized body pain and fatigue due to comorbidities and a small tear in the skin of the lower limb; in the LE group, four consisted of an exacerbation of comorbidity and one of chronic knee pain when walking.

McNamara (2015) [21] described that in the EG, 80% preferred to carry on with WE, 10% preferred LE and only 10% did not want to train either way, while in LE, 55% preferred to carry on with the activity (land exercise), 25% did not want to participate and 20% showed a preference for WE.

#### 3.4.2. WE versus CG without Therapy or Usual Medical Care

Three studies found a significant improvement in the EG in functional capacity measured by 6MWT or 30″SST and a significant difference between the EG and the CG [24,27,28], while De Souto Araujo [22] found a significant improvement in dyspnea index for the EG, and a worsening in CG.

Using an incremental test limited by clinical symptoms on a cycloergometer and the ISWT, two studies [24,27] observed that the EG improved significantly, while the CG did not. 

Two studies [22,25] showed a significant improvement in MIP and MEP for the EG. Three studies [22,25,27] found significant differences in the EG with respect to the CG in terms of MIP. 

Regarding pulmonary function, De Souto Araujo [22] found a significant improvement in FEV1 in the EG, and a significant decrease in the Tiffeneau index in the CG. The study by Wu [25] found no significant differences.

Using ESWT, McNamara (2013) [27] showed a significant improvement in the EG and compared to the CG.

Two studies that evaluated HRQOL using the SGRQ [22,24] showed improvements in all domains in the EG with respect to the CG, with statistically significant differences. In addition, Gallo-Silva [28] found a significant decrease in the domains symptoms, impact and total score in the CG. McNamara (2013) [27] with the CRDQ observed significant differences between the EG and the CG in the domains of dyspnea and fatigue.

McNamara (2015) [21] collected information about preferences of the CG without intervention, indicating that 53% preferred the aquatic environment, 43% preferred the land environment, and 4% did not prefer either form.

#### 3.4.3. Synthesis of Results

To directly observe the effects of WE on the different outcome measures, a table has been prepared describing whether there were positive effects, no effects or negative effects after the intervention for each of the outcome measures for each study (Table 3).

### 3.5. Risk of Bias of Studies Included and Meta-Analysis

Table 4 summarizes the methodological quality of the eight studies in this review.

Six have moderate methodological quality, with scores between 6 and 8 on the PEDro scale [21,23,24,25,26,27], and two studies have low quality with scores between 4–5 [22,28]. No studies blinded participants or therapists.

**Figure 2:**
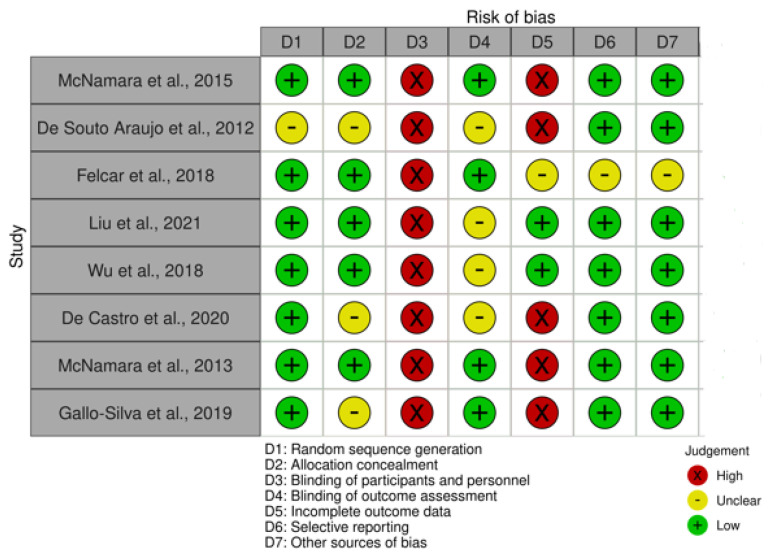
Risk of bias assessment according to the Cochrane Collaboration Risk of Bias Tool [21,22,23,24,25,26,27,28,30].

Either a high risk of bias was observed using the Cochrane Collaboration Risk of Bias tool (Figure 2) in some domains of the study design, or information is missing so it was not possible to reach a conclusion about risk.

Within selection risk, the randomization process was well reported by all trials, except for the study by De Souto Araujo [22]. Allocation concealment was generally low risk, although three studies did not report enough to draw a conclusion [22,26,28]. All of the studies have a high risk of performance bias (concealment of participants and personnel). Four of the trials have a low risk of detection [21,23,27,28]. Regarding attrition bias, five of the eight studies have a high risk of bias [21,22,26,27,28]. All trials have a low risk of reporting bias and other biases, with the exception of the Felcar trial [23], which underreported in both domains.

**Figure 3:**
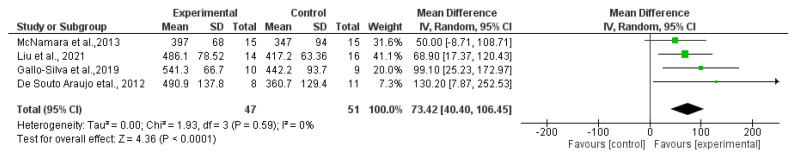
Meta-analysis based on the results of the 6MWT comparing WE with CG [22,24,27,28].

A meta-analysis was conducted on functional capacity, measured via the 6MWT in four clinical trials [22,24,27,28], comparing WE with CG (Figure 3). As can be seen in the forest plot, the samples of the studies would be similar with zero heterogeneity, and the results were statistically significant in favor of the EG (*p* < 0.0001). That is, the functional capacity in the EG was superior to the CG in patients with COPD (SMD: 73.42; IC95%: 40.40–106.45; I^2^: 0%).

**Figure 4:**
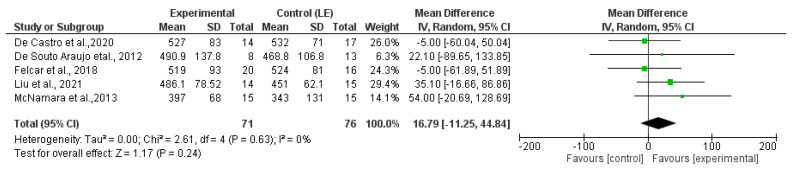
Meta-analysis based on the results of the 6MWT comparing WE with LE [22,23,24,26,27].

When comparing the functional capacity of the WE with the LE from five clinical trials [22,23,24,27,28], measured with the 6MWT (Figure 4), no statistically significant differences were found between the two groups (*p* = 0.24).

**Figure 5:**
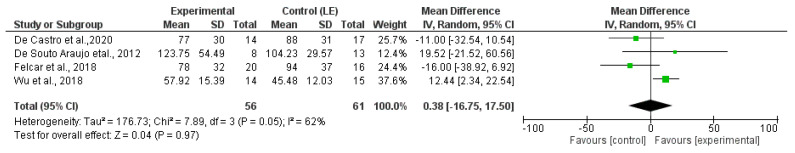
Meta-analysis based on the results of the MIP comparing WE with LE [22,23,25,26].

Four clinical trials [22,23,25,26] compared respiratory muscle strength in the WE with the LE, measured via MIP (Figure 5). The results of this meta-analysis were not statistically significant (*p* = 0.97), and therefore, there was no difference in favor of one intervention group or the other.

**Figure 6:**
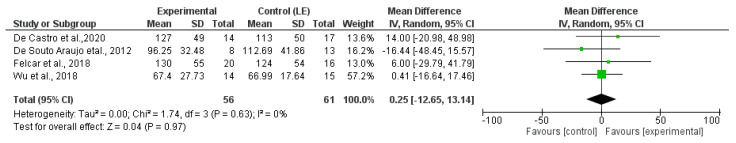
Meta-analysis based on the results of the MEP comparing WE with LE [22,23,25,26].

Four clinical trials measured respiratory muscle strength [22,23,25,26] via MEP, comparing the WE with the LE (see Figure 6), yet they did not provide statistically significant differences in favor of one group or the other (*p* = 0.97).

Since the quality-of-life variable was not measured with a similar questionnaire in at least 4–5 of the studies included, a meta-analysis was impossible to conduct.

## 4. Discussion

This systematic review with meta-analysis has analyzed the effectiveness of WE to improving functional capacity and respiratory muscle strength in people with COPD in RCTs over the last ten years.

Functional capacity was measured in six of the eight studies by the 6MWT [22,23,24,26,27,28], all of which found a significant improvement in the training groups, both in water and on land. However, the CGs did not obtain improvements [24,27,28] or even worsened their functional capacity [22]. Moreover, in the study by Liu [24], the 30″SST was also utilized, and the results coincided with those of the 6MWT. However, the result of the meta-analysis reveals that there is not enough evidence to confirm that WE is better than LE, although it is corroborated with respect to CG. Previous studies and reviews [2,3,15,31] have agreed on this statistically significant improvement in functional capacity following the WE compared to the CG, although they also do not find significant improvements when comparing WE with LE. Previous studies have observed that the improvement in exercise capacity is related to an increase in muscle strength [2,32,33,34]. The studies that evaluated peripheral muscle strength in our review also showed an improvement in functional capacity [23,26].

The results of this review regarding maximal exercise capacity show a significant improvement in both the WE and the LE compared to the CG, coinciding with the results of the review by McNamara [3], while the review by Chen [2] found no improvement in any of the intervention groups. The improvement in functional capacity and maximal exercise capacity after performing WE could be due to the greater training stimulus received in the aquatic environment, since each movement is resisted by the hydrostatic pressure and the turbulence effect of the water, which translates into better results in the 6MWT, 30″SST and ISWT tests [3,4].

Due to the load of water exercise on the respiratory muscles, there may be a significantly positive effect on their strength [2,16]. Five studies assessed respiratory muscle strength using MIP and MEP [22,23,25,26,27], and only the study by McNamara (2013) [27] found no improvement in either group; the remaining studies showed improvement in both the WE and the LE. In Felcar [23] the improvement of MIP in the WE occurred at 3 months, while in the LE, it took place at 6 months. In De Castro [26], the improvement of MEP in the WE was greater than in the LE. The studies with the CG showed that this group did not improve [22,27] or even worsened [25]. After performing the meta-analysis on these measures, it was found that there is not enough evidence to confirm the results. Nevertheless, several studies have pointed out that a therapeutic intervention in water may provide greater benefits than similar exercise training on land [15,31].

Only three studies assessed pulmonary function [22,23,25], with two finding no significant change [23,25], although De Souto Araujo [22] shows a significant improvement in FEV1 in the WE and the LE, and a decrease in the Tiffeneau index in the CG. The data collected in this study are similar to those collected in the review by Chen [2], where there were no significant statistical findings to affirm that aerobic exercise improves pulmonary function in patients with COPD, although it indicates that in studies with durations of more than 8 weeks of intervention, there is a trend towards improvement. This is controversial considering the data collected in this review, since one study had a duration of 24 weeks [23], another 12 weeks [25] and, the study by De Souto Araujo [22], which was the only one that found a significant improvement in pulmonary function, lasted 8 weeks. From the articles included here, it can be deduced that perhaps the type of exercise and the condition of the patients at the beginning of the study may be determining factors for this significant improvement or only the maintenance of the individual’s capacity (for example, in patients with a very low FEV1). In previous studies, such as Martín-Valero [15], it has been found that the WE intervention is probably more favorable for patients with lower functional capacity.

Dyspnea is one of the main symptoms among people suffering from COPD, appearing both on exertion and at rest, and causes physical deconditioning leading to a more sedentary lifestyle, reducing physical function, decreasing respiratory and peripheral muscle mass and reducing quality of life [4,5,6,7]. Five studies assessed HRQOL, three of which used the SGRQ [22,24,28], generally showing an improvement in the WE and the LE in all of them, while two studies found a worsening in the total CG score [22,24]. Only Gallo-Silva [28] found that the total score and other domains improved in CG. Two studies assessed this measure using the CRDQ, finding in the study by Felcar [23] an improvement in the WE and the LE, while the study by McNamara (2013) [27] only found a significant improvement in the WE in the fatigue domain, and a significant difference in the WE compared to the CG in the dyspnea and fatigue domains. Previous studies [2,3,35] have shown similar results at the same exercise intensity, heart rate and blood lactic acid. Similarly, the perception of fatigue and dyspnea improved in both the WE and the LE, although perception of fatigue improved more following WE, indicating that patients can reach and even overcome the required exercise intensity in less time in an aquatic environment [2,3,35]. This is especially beneficial for weak athletes or elderly people [2,35], as it makes aquatic therapy a therapeutic option.

The main part of the physical training performed in the trials included in this review was aerobic, strength, concomitant and Liuzijue exercise, carried out with an intensity of 3 to 6 according to the modified Borg scale for dyspnea and perceived exertion. These varied interventions address different aspects of physical and respiratory fitness in these patients, and have contributed to improvements in functional capacity, respiratory muscle strength and quality of life. Previous studies in COPD patients affirm that strength and aerobic physical exercise are beneficial and should be included in training programs [31,36,37]. It was also observed that there are different levels of immersion in water (umbilicus or xiphoid-clavicle appendage) which may have influenced the resistance to movement and the distribution of workload in the musculoskeletal and respiratory system. In the studies included, the water temperature ranged from 26 °C to 34 °C, and there was much disparity between the characteristics of the aquatic environment, such as depth, width and length of the pool, which may have provided different stimuli and challenges to the participants during WE intervention. This may explain, in part, why some studies found greater or faster improvements in the WE than in the LE or CG.

Water temperature has been described as a very influential factor in the physiological benefits obtained in the body during aquatic therapy [4]. When water temperature is higher than 28 °C, improvements in O_2_ transport are observed due to increased cardiac output, resulting from the combination of hydrostatic back pressure and body warming [38]. In addition, hydraulic pressure elevates the diaphragm and facilitates expiration, decreasing dead space in the lungs [39,40]. The combination of hydrostatic pressure and water temperature can induce an increase in cardiac output, reduce sputum viscosity and increase respiratory rate, resulting in an improved gas exchange rate in the lungs [2,38]. These effects may be especially beneficial for the improvement of respiratory and circulatory function during aquatic therapy in people with respiratory diseases.

The physiotherapist/patient ratio also ranged from one physiotherapist for every 2 patients to one physiotherapist for every 13 patients. The benefits of group therapy in COPD patients have been observed in several studies [31,41], as it provides mutual support and empowerment to patients, improving symptoms and psychosocial effects, in addition to being more cost-effective by bringing several patients together under the supervision of one physiotherapist [31,41].

Many COPD patients are elderly and may have other comorbidities such as arthritis, obesity or other musculoskeletal or neurological problems that may affect their exercise capacity, although it has been seen and proven that the unique properties of water can enable them to exercise at a higher intensity by decreasing the impact of exercise on their comorbidity [3,39,42]. Also, WE is a safe treatment method in a wide variety of pathologies, as it does not cause serious adverse events, for example, in arthritis [43,44], fibromyalgia [45], and neurological disorders such as Parkinson [46] and multiple sclerosis [47] and asthma [48]. Regarding adverse effects in this systematic review, three studies observed no adverse effects [24,25,26], and two other studies found three adverse effects in the EG: a small skin tear in a lower limb, generalized pain, and fatigue due to comorbidities [21,27].

WE has been shown to decrease pain, improve physical function, improve sleep quality and reduce fatigue levels [43,44,45]. In addition, it facilitates muscle control, coordination and mobility [46,47], contributing to motor and functional recovery without significant adverse effects. This allows us to affirm that physical exercise in water is completely safe for people with COPD, even in those with comorbidities.

The trial by McNamara (2015) [21] reported WE training preferences of participants with comorbid physical conditions, such as obesity or unfavorable orthopedic conditions, which limited their functional capacity on land, logically making water-based exercise much more beneficial. Further research including this variable is needed to understand the preference of people with COPD.

In previous reviews such as Chen [2], it was observed that aerobic exercise, performed on land or in water, significantly improves functional capacity, exercise endurance and dyspnea in COPD patients. This is due to the fact that aerobic exercise is capable of improving the body’s oxidation capacity, the vital capacity of these patients and their cardiopulmonary adaptability, as well as reducing dynamic hyperinflation [2,40]. Our review supports that physical exercise in general, whatever the modality, is able to improve these characteristics in COPD patients.

This review shows that the studies whose results found no significant differences coincide with those that described physical comorbidities [27], the existence of smokers among their participants [21,27], or those whose mean FEV_1_ was lower than the rest [22,23,27,28], meaning these covariates may have influenced in the obtained results.

The methodological quality of the studies included was mostly moderately good, although it should be considered that, due to the type of intervention, participants and therapists could not be blinded. The risk of bias lies in the lack of information provided by some studies on outcome measures.

The limitations of this systematic review include the lack of studies with high methodological quality and low risk of bias; specifically, there were two articles with low methodological quality and high risk of bias [22,28], so the quality of the evidence may be compromised, affecting the reliability of the conclusions and their clinical utility. Moreover, the studies include small samples, variety in the characteristics of the participants and the aquatic environment, in the duration of the sessions and studies, and in the type of exercise performed. These issues make it difficult to reach a consensus on the results and increase the risk of bias in this review. All these factors, together with the small number of articles on the subject and the lack of certain information, have limited the possibility of carrying out a more powerful meta-analysis of all the studies in this review. Also, potential limitations of this study include the exclusion of former but still valuable research, particularly when studying long-term outcomes. Additionally, emerging interventions may have limited data within the specified time frame.

Careful consideration should be given in future studies to the choice and standardization of aquatic training protocols to facilitate comparison and generalization of findings to the COPD population. Furthermore, these interventions should be longer term, with repeated assessments, to determine whether the process of improvement in any outcome measure is accelerated by WE. Other measures such as balance, adverse effects and patient preferences would also need to be included in the assessment, as the number of studies collecting this information is too few to prove the efficacy of WE in people with COPD.

It is evident from our analysis that there is a significant variation in the response to aquatic interventions among COPD patients. Some studies have reported substantial improvements in functional capacity, respiratory muscle strength, and quality of life following WE, while others have shown more modest or even non-significant effects. We believe that this variability could be attributed to several factors, including the presence of physical comorbidities, the smoking status of participants and the baseline lung function.

Future studies should aim to delve deeper into these factors to better understand why certain COPD patients may benefit more from WE than others. This would involve stratifying participants based on their comorbid conditions, such as obesity or orthopedic issues, which might limit their ability to perform land-based exercises effectively. Additionally, investigating the preferences of COPD patients with specific comorbidities, as highlighted in the study by McNamara (2015) [21], could offer valuable insights into tailoring interventions to individual needs.

Moreover, it is crucial to conduct more extensive research with larger sample sizes to provide a more comprehensive analysis of how patient characteristics and comorbidities interact with the effectiveness of aquatic interventions. This would help in developing personalized exercise regimens for COPD patients, ensuring that they receive the most suitable and beneficial therapy based on their unique profiles.

Among the advantages of WE are that the buoyancy of water reduces impact on joints and muscles, making it easier for patients to move and exercise; resistance of water can provide a challenging workout, improving strength and endurance; hydrostatic pressure of water can reduce swelling and improve circulation; and aquatic therapy can be enjoyable, increasing motivation and adherence to treatment [3,47,48]. On the other hand, there are also some possible disadvantages of WE: some medical conditions, like open wounds or infections, may constitute contraindications; effectiveness depends on the skill and experience of the therapist and the specific exercises used [47].

In the case of LE, some patients may find these exercises more accessible and convenient based on their baseline physical activity levels [45]. However, LE has also some disadvantages, since chronic joint pain [43], skeletal muscle dysfunction [45,46,47], and imbalance [10] may limit the effectiveness of land-based exercises; moreover, a lack of interest and poor adherence to land-based exercises for some patients mean that as their disease condition worsens, they are unable to complete the established intervention program [2].

It is important to note that the choice between WE and LE should be based on individual preferences, physical condition and specific therapeutic goals [45,48]. Consulting with a healthcare provider is essential before starting any new exercise program, especially for individuals with medical conditions [48].

## 5. Conclusions

This systematic review with meta-analysis supports, thanks to the qualitative analysis, the positive impact of WE on key outcomes, including functional capacity, maximum exercise capacity, respiratory and peripheral muscle strength, and quality of life (particularly in domains related to dyspnea and fatigue). Importantly, this study did not identify statistically significant differences between WE and LE in terms of their effectiveness in achieving these improvements.

There are significant differences in favor of the WE group versus a non-exercising control group in terms of the reported benefits in functional capacity.

Exercise in the aquatic environment provides greater stimuli that lead to an improvement in exercise capacity and maximal exercise capacity, as well as an improvement in the respiratory musculature due to the load of the water on it.

WE provides benefits due to the unique properties of water, such as reduced impact on comorbidities and enhanced exercise intensity without causing significant adverse events. It is considered safe for individuals with COPD, especially in patients with comorbidities.

After the analysis of the included studies, it has been observed that perhaps the condition of the patients at the beginning of the studies and the type of exercise performed may influence the improvement of the outcome measures, so future studies should study them in depth, stratifying the patients according to their initial characteristics or comorbid conditions.

## Figures and Tables

**Table 1:** Search strategy and article selection.

Databases	Search Strategy	Results	Criteria	Selection of Articles
PubMed	Aquatic therapy AND Chronic obstructive pulmonary disease	7	− RCT − 2012–2023	− McNamara et al., 2015 [21] − De Souto Araujo et al., 2012 [22]
	Aquatic exercise AND Chronic obstructive pulmonary disease	4	− RCT − 2012–2023	
	Water-based exercise AND Chronic obstructive pulmonary disease	21	− RCT − 2012–2023	− Felcar et al., 2018 [23] − Liu et al., 2021 [24] − Wu et al., 2018 [25] − De Castro et al., 2020 [26] − McNamara et al., 2013 [27] − Gallo-Silva et al., 2019 [28]
PEDro	Aquatic therapy AND Chronic obstructive pulmonary disease	1	− RCT − 2012–2023	
	Aquatic exercise AND Chronic obstructive pulmonary disease	2	− RCT − 2012–2023	
	Water-based exercise AND Chronic obstructive pulmonary disease	5	− RCT − 2012–2023	
Scopus	Aquatic therapy AND Chronic obstructive pulmonary disease	6	− RCT − 2012–2023	
	Aquatic exercise AND Chronic obstructive pulmonary disease	9	− RCT − 2012–2023	
	Water-based exercise AND Chronic obstructive pulmonary disease	23	− RCT − 2012–2023	
Web of Science	Aquatic therapy AND Chronic obstructive pulmonary disease	20	− RCT − 2012–2023	
	Aquatic exercise AND Chronic obstructive pulmonary disease	8	− RCT − 2012–2023	
	Water-based exercise AND Chronic obstructive pulmonary disease	28	− RCT − 2012–2023	

RCT: randomized controlled trial.

**Table 2:** Summary of the characteristics of the selected studies.

RCTs	Participants	Water Exercise Intervention(s)	Comparison Intervention(s)	Aquatic Environment	Treatment Duration	Variables Measures	Results
McNamara, 2015 [21]	N = 45 Mean age: 72 years; minimum and maximum (61–82) 58.49% women 41.51% men FEV_1_ % predicted mean: 59; minimum and maximum (35.78) Presence of smokers. With comorbidities.	Experimental group (EG) (n = 15): Training session with aerobic exercises (walking, jogging, jumping, cycling, etc.) in water for upper limbs (ULs) and lower limbs (LLs). Intensity 3–5 modified Borg scale. Individual progression. Immersion level between xiphoid appendages and clavicle.	− Control group (CG) (n = 15): No exercise session. − Land exercise intervention group (LE) (n = 15): Training session with aerobic exercise (walking, jogging, jumping, cycling, etc.) on land of ULs and LLs. Intensity 3–5 on modified Borg scale. Individual progression.	− Length: 18 m. − Width: 6 m. − Depth: 1.1–1.6 m. − Tª: 34 °C pool, 30 °C environment. − Relative humidity: 30%. − Swimming pool entrance: ramp, stairs and hoist. − Sanitation: sodium hyperchloride + ultraviolet light with diatomaceous earth as filtration method. − Physiotherapist/participant ratio of 1:10.	60′, 3 times/week, for 8 weeks.	− Transport assistance. − Adverse effects and withdrawal. − Enjoyment using a 5-point Likert scale. − Satisfaction with aquatic environment. − Favorable factors: personal support, enjoyment, sense of achievement, noticeable improvements, personal motivation and support from other participants. − Barriers. − Preference of physical training environment.	− Transport assistance: 1 EG and 4 LE. − Adverse effects and withdrawal: Three from EG did not complete the program: 1 x small skin tear in lower limb and 2 x general body pain and fatigue x comorbidities. Five from LE did not complete program: 4 x exacerbation of physical comorbidity and 1 x chronic knee pain when walking. − Enjoyment: > in EG. − Satisfaction with aquatic environment: 18/18 satisfied LE. − Favorable factors: EG and LE = in personal support and personal motivation, EG > all other factors. − Barriers: not in EG. − Preference: EG 16/18 aquatic environment and 2/18 gym. LE 11/20 gym, 4/20 aquatic and 5/20 no mode.
De Souto Araujo, 2012 [22]	N = 32 Mean age: 63 years; minimum and maximum (49–81) 37.5% Women 62.5% Men FEV_1_ % predicted mean: 43; minimum and maximum (28.58) No smokers. No comorbidities or exacerbations.	EG (n = 8): First stage: warm-up continuous calisthenic exercises ULs and LLs 15’ associated with breathing. Second-stage: ULs training with weights and diagonal movements of 2’ and 2’ of rest. Third stage: LLs training with floats on legs and cycling movement 30’. Fourth stage: cool down 15’. Individual progression.	− CG (n = 11): No exercise session. − LE (n = 13): First stage: 15’ continuous calisthenic exercises associated with breathing. Second stage: ULs exercises with weights and diagonal movements 2’ and 2’ rest. Third stage: LLs training, stationary bike 30’. Fourth stage: cool down 15’. Individual progression.	− Tª water: 30–34 °C.	90′, 3 times/week, for 8 weeks.	− Anthropometry (weight, height, BMI). − Pulmonary function via forced vital capacity (FVC), forced expiratory volume in 1 s (FEV1) and Tiffeneau index (FEV1/FVC). − Respiratory muscle strength via maximal inspiratory pressure (MIP) and maximal expiratory pressure (MEP). − Functional capacity via 6’ walk test (6MWT). − BODE mortality predictor index. − Health-related quality of life (HRQOL) using modified St. George’s Hospital Respiratory Disease Questionnaire (SGRQ).	− Pulmonary function: FEV_1_ improves in EG and LE. CG < FEV_1_/FVC. − Respiratory muscle strength: MIP and MEP improved in EG and LE. − Functional capacity: LE < perceived exertion rate. Dyspnea index < in EG and > in CG. − BODE: < in EG and LE and > in CG. − HRQOL: < LE in all domains, and > in CG domains impact and total score.
Felcar, 2018 [23]	N = 36 Mean age: 69 years; minimum and maximum (60–78) 36.1% women 63.9% men FEV_1_ % predicted mean: 47; minimum and maximum (31.65) Presence of smokers. No comorbidities or exacerbations in last 3 months.	EG (n = 20): water training session with warm-up (1’ walk and metabolic exercises ULs), aerobic exercises (cycling and walking with rhythm according to sound stimuli), strength training LLs (quadriceps) and ULs (biceps and triceps) and stretching LLs, ULs, cervical and trunk. Individual load. Umbilical immersion level.	LE (n = 16): land training session with warm-up (1’ walk and metabolic exercises ULs), aerobic exercises (cycling and walking with rhythm according to sound stimuli), strength training LLs (quadriceps) and ULs (biceps and triceps) and stretching LLs, ULs, cervical and trunk. Individual load.	− Length: 12.7 m. − Width: 7.1 m. − Depth: 1 m. − Tª water: 33 °C. − Swimming pool entrance: ramp and stairs. − Sanitation: chlorine. − Physiotherapist/participant ratio of 2:4.	60′ in the afternoon 3 times/week for the first 12 weeks, and 2 times x week for the last 12 weeks.	− Physical Activity Daily Living (PADL) using Power Walker-PW610 activity monitor. − Pulmonary function via spirometry. − Peripheral muscle strength via 1 repetition maximum (1RM) test of quadriceps, biceps and triceps brachii. − Respiratory muscle strength via MIP and MEP. − Body composition via bioimpedance. − Maximal exercise capacity via incremental shuttle walking test (ISWT). − Functional exercise capacity via 6MWT. − HRQOL via Chronic Respiratory Disease Questionnaire (CRDQ). − Functional status using London Chest Activity of Daily Living (LCADL) scale. − Activities of Daily Living (ADL) limitations due to dyspnea using the Medical Research Council (MRC) scale. − Anxiety and depression symptoms using the Hospital Anxiety and Depression Questionnaire (HADQ). − BODE mortality predictor index. − Comorbidities using a specific questionnaire.	− PADL: > EG and LE. At baseline, 8/36 (22%) physically active (4/16 LE and 4/20 EG). After 6 months, 15/36 (42%) physically active (5/16 LE and 10/20 EG). − Peripheral muscle strength: > EG and LE after 3 months. − Respiratory muscle strength: > MIP EG after 3 months and LE after 6 months. > MEP EG and LE after 3 months. − Maximal exercise capacity: > EG at 3 months and LE at 6 months. − Functional exercise capacity: > EG and LE after 3 months. − HRQOL: < in EG and LE after 3 months. − Functional status: improvement in EG after 3 months and LE after 6 months. − Dyspnea-related limitations in ADLs: < EG at 3 months. − Comorbidities: 75% (12/16) LE and 65% (13/20) EG. − EG had higher effect size values for PADL, peripheral muscle strength, exercise capacity, HRQOL and functional status.
Liu, 2021 [24]	N = 45 Mean age: 65 years; minimum and maximum (54–76) 26.7% women 73.3% men FEV_1_ % predicted mean: 57; minimum and maximum (37.81) No exacerbation in the last month and no severe comorbidities.	EG (n = 14): Liuzijue session in water divided into 3 parts: 1st, warm-up 5–10’ stretching and joint mobility; 2nd, Liuzijue exercise 45’; and 3rd, cool-down 5–10’ regulating breathing, relaxing and stretching muscles. Intensity 4–6 on modified Borg scale for dyspnoea and perceived exertion. Immersion level between xiphoid appendix and clavicle.	− CG (n = 16): No exercise session. − LE (n = 15): Liuzijue session on land divided into 3 parts: 1st, warm-up 5–10’ stretching and joint mobility; 2nd, Liuzijue exercise 45’; and 3rd, cool down 5–10’ regulate breathing, relax and stretch muscles. Intensity 4–6 on modified Borg scale for dyspnea and perceived exertion.	− Tª: 26–30 °C pool, 30–34 °C environment. − Relative humidity: 60–80%. − Sanitation: chlorine.	60′, 2 times/week, for 12 weeks.	− Maximal exercise capacity via a cycloergometer with gas exchange measurement. − Functional exercise capacity via 6MWT and the 30″ stand up test (30″ SST). − HRQOL via SGRQ.	− Maximal exercise capacity: VO2 max > EG. Maximum work rate (W max) > in EG and LE. − Functional exercise capacity: for 6MWT > in EG and LE and EG > CG. 30″ SST > in EG and LE. − HRQOL: < all domains in EG and LE and > domain CG activity score. EG < CG and LE < CG in all domains. − Adverse events: no adverse events observed.
Wu, 2018 [25]	N = 45 Mean age: 65 years; minimum and maximum (54–76) 26.7% women 73.3% men FEV_1_ % predicted mean: 57; minimum and maximum (37.81) No exacerbation in the last month and no severe comorbidities.	EG (n = 14): Liuzijue exercise session in water with 3 parts: 1st, warm-up with stretching of trunk and limbs 10’; 2nd, Liuzijue exercise 40’; and 3rd, cool-down with regular breathing and muscle relaxation 10’. Intensity 4–6 on modified Borg scale for dyspnoea and perceived exertion. Immersion level between xiphoid appendix and clavicles.	− CG (n = 16): No exercise session. − LE (n = 15): Liuzijue exercise session on land with 3 parts: 1st, warm-up with trunk and limbs stretching 10’; 2nd, Liuzijue exercise 40’; and 3rd, cool down with breathing regulation and muscle relaxation 10’. Intensity 4–6 on modified Borg scale for dyspnea and perceived exertion.	− Tª water: 30–34 °C.	60′, 2 times/week, for 12 weeks.	− Pulmonary function via spirometry. − Respiratory muscle strength via MIP and MEP. − Muscle strength ULs (elbow flexion and extension) and LLs (knee flexion and extension) using CON-TREX isokinetic dynamometer.	− Respiratory muscle strength: MIP and MEP > in EG and LE. MEP < in CG. MIP and MEP EG and LE > CG. − ULs muscle strength: total work (TW) flexor and extensor > in EG and LE. TW flexor LE > CG, while TW extensor EG > CG. Resistance ratio (RR) flexor and extensor < in CG. RR EG and LE > CG. − Muscle strength LLs: EG and LE > peak torque (PT) extensor and peak torque/bodyweight (PT/BW), while PT flexor < in CG. PT and PT/BW EG > CG. TW extensor > in EG and LE. TW flexor > in EG, while < in CG. TW flexor and extensor and RR EG > CG, and only TW flexor LE > CG. − Adverse events: none observed.
De Castro, 2020 [26]	N = 31 Mean age: 65 years; minimum and maximum (56–73) 41.9% women 58.1% men No severe comorbidities and no exacerbations 3 months earlier.	EG (n = 14): water training session with warm-up (1’ walk and calisthenics), aerobic training (cycling or walking according to sound rhythm), strength training LLs (quadriceps) and ULs (biceps and triceps), and stretching ULs, LLs and trunk muscles. Intensity 4–6 on modified Borg scale. Umbilical immersion level.	LE (n = 17): training session with warm-up (1’ walk and calisthenics), aerobic training (cycling or walking according to sound rhythm), strength training LLs (quadriceps) and ULs (biceps and triceps), and stretching ULs, LLs and trunk muscles. Intensity 4–6 on modified Borg scale.	− Length: 12.7 m. − Width: 7.1 m. − Depth: 1 m. − Tª water: 33 °C.	60′, 3 times/week, for 12 weeks.	− Static balance by using a force platform. − Functional balance using up and go test (UGT). − Functional exercise capacity via 6MWT. − Maximal exercise capacity via ISWT. − Respiratory muscle strength via MIP and MEP. − Peripheral muscle strength via isometric quadriceps dynamometer.	− Functional balance: > EG. − Functional exercise capacity: > EG and LE. − Maximal exercise capacity: > EG and LE. − Respiratory muscle strength: > EG and LE, but EG > MEP than LE. − Peripheral muscle strength: > EG and LE. − Adverse events: none observed.
McNamara, 2013 [27]	N = 45 Mean age: 72 years; minimum and maximum (61–82) 58.49% women 41.51% men FEV_1_ % predicted mean: 59; minimum and maximum (35.78) Presence of smokers. With physical comorbidities.	EG (n = 15): Water training session with aerobic exercise (walking, jogging, jumping, cycling, etc.); 8’ warm up, 20’ aerobic exercise LLs, 3’ rest, 15’ aerobic exercise LLs, 2’ rest, 10’ aerobic exercise ULs and 2’ cool-down. Intensity 3–5 on the modified Borg scale. Individual progression. Immersion level between xiphoid appendages and clavicle.	− CG (n = 15): No exercise session. − LE (n = 15): Land training session with aerobic exercises (walking, jogging, jumping, cycling, etc.); 8’ warm up, 20’ aerobic exercise LLs, 3’ rest, 15’ aerobic exercise LLs, 2’ rest, 10’ aerobic exercise ULs and 2’ cool-down. Intensity 3–5 on the modified Borg scale. Individual progression.	− Length: 18 m. − Width: 6 m. − Depth: 1.1–1.6 m. − Tª: 34 °C pool, 30 °C environment. − Relative humidity: 30%. − Physiotherapist/participant ratio of 1:10.	60′, 3 times/week, for 8 weeks.	− Aerobic exercise capacity via endurance shuttle walking test (ESWT). − Pulmonary function via spirometry (FEV_1_ and FVC), and body plethysmography (CO diffusing capacity). − Respiratory muscle strength via MIP and MEP. − Exercise capacity via 6MWT and ISWT with external rhythm. − HRQOL via CRDQ. − Anxiety and depression via HADQ.	− Adverse effects and withdrawal: Three EG did not complete program: 1 x small x lower and 2 x general body pain and fatigue x comorbidities skin member. Five LE did not complete program: 4 x physical comorbidity and 1 x knee pain chronic walking. − Exercise capacity: 6MWT > EG and LE. EG > CG and LE > CG. ISWT and ESWT > in EG. EG > ISWT and ESWT compared to LE and CG. − Respiratory muscle strength: MIP EG > CG. − HRQOL: in fatigue domain. EG < CG in fatigue and dyspnea domains.
Gallo-Silva, 2019 [28]	N = 19 Mean age: 66 years; minimum and maximum (57–76) 0% women 100% men FEV_1_ % predicted mean: 48, minimum and maximum (21.74) No exacerbations in previous 3 months and no severe comorbidities.	EG (n = 10): usual medical treatment and interval aerobic training: 10’ warm-up with stretching, calisthenics and low intensity exercises; 20–40’ aerobic exercises with trunk and limbs divided into 6 stages with 1’ rest between each one; and 10’ cool-down with stretching and breathing exercises. Intensity 4–6 modified Borg scale.	CG (n = 9): received usual medical care, without exercise.	− Length: 18 m. − Width: 6 m. − Depth: 1.1–1.6 m. − Tª water: 32 °C. − Relative humidity: 30%. − Physiotherapist/participant ratio of 1:12.	60′, 3 times/week, for 8 weeks.	− Pulmonary function via spirometry. − Heart rate variability (HRV) via monitor. − HRQOL via SGRQ. − Functional exercise capacity via 6MWT.	− HRV: global improvement EG. EG better than CG. − HRQOL: > in symptom, impact and total domains in CG. EG < CG in all domains. − Functional exercise capacity: > EG. EG > GC. − Calculation of the effect between CG and EG showed that EG > training effect (>0.8) compared to CG.

<: decrease; >: increase; 1RM: 1 repetition maximum; 30″ SST: 30″ stand-up test; 6MWT: 6 min walk test; AT: anaerobic threshold; BMI: body mass index; BW: body weight; CG: control group; CO: carbon monoxide; CRDQ: chronic respiratory disease questionnaire; EG: experimental group; h: hour/s; ESWT: endurance shuttle walk test; FEV_1_: forced expiratory volume in first second; FVC: forced vital capacity; HADQ: hospital anxiety and depression questionnaire; HRQOL: health-related quality of life; HRV: heart rate variability; ISWT: incremental shuttle walk test; LCADL: London Chest Activity of Daily Living scale; LE: Land Exercise Intervention Group; LLs: lower limbs; m: meter; MCID: minimum clinically important difference; MEP: maximal expiratory pressure; MIP: maximal inspiratory pressure; MRC: Medical Research Council scale; N^o^.: Number; °C: degrees Celsius; PADL: physical activity in daily life; PT: peak torque; RCT: randomized clinical trial; RR: resistance ratio; SGRQ: modified St George’s Hospital Respiratory Diseases Questionnaire; Tª: temperature; TW: total work; UGT: up and go test; ULs: upper limbs; VO2max: maximal oxygen volume; Wmax: maximal work rate; x: due to.

**Table 3:** Synthesis of results.

RCTs	Functional Exercise Capacity	Maximum Exercise Capacity	Respiratory Muscle Strength	Pulmonary Function	Aerobic Exercise Capacity	Peripheral Muscle Strength	HRQOL	BODE Index	Symptoms of Anxiety and Depression	Static and Functional Balance	Adverse Effects	Preferences
McNamara, 2015 [21]	N/A	N/A	N/A	N/A	N/A	N/A	N/A	N/A	N/A	N/A	!	+
De Souto Araujo, 2012 [22]	+	N/A	+	+	N/A	N/A	-	+	N/A	N/A	N/A	N/A
Felcar et al., 2018 [23]	+	+	+	-	N/A	+	+	-	-	N/A	N/A	N/A
Liu, 2021 [24]	+	+	N/A	N/A	N/A	N/A	+	N/A	N/A	N/A	-	N/A
Wu, 2018 [25]	N/A	N/A	+	-	N/A	+	N/A	N/A	N/A	N/A	-	N/A
De Castro, 2020 [26]	+	+	+	N/A	N/A	+	N/A	N/A	N/A	+	-	N/A
McNamara, 2013 [27]	+	+	-	N/A	+	N/A	-	N/A	-	N/A	!	N/A
Gallo-Silva, 2019 [28]	+	N/A	N/A	N/A	N/A	N/A	-	N/A	N/A	N/A	N/A	N/A

!: negative significant results for EG; -: no significant results for EG; +: positive significant results for EG; N/A: not applicable.

**Table 4:** Evaluation of the methodological quality of the articles included in this report.

	PEDro Scale Items [29]	Specified Selection Criteria	Random Allocation	Concealed Assignment	Similar Groups	Blinded Subjects	Blinded Therapists	Blinded Assessors	Adequate Follow-Up	Intention-to-Treat Analysis	Results of Group Comparisons	Point Measurements of Variability	Total Score
RCTs													
McNamara, 2015 [21]		1	1	1	1	0	0	1	1	0	1	1	7
De Souto Araujo, 2012 [22]		0	1	0	1	0	0	0	0	0	1	1	4
Felcar, 2018 [23]		1	1	1	1	0	0	1	0	0	1	1	6
Liu, 2021 [24]		1	1	1	1	0	0	1	1	0	1	1	7
Wu, 2018 [25]		1	1	1	1	0	0	1	1	0	1	1	7
De Castro, 2020 [26]		1	1	1	1	0	0	1	0	0	1	1	6
McNamara, 2013 [27]		1	1	1	1	0	0	1	1	0	1	1	7
Gallo-Silva, 2019 [28]		0	1	1	1	0	0	0	0	0	1	1	5

RCT: randomized controlled trial; 1: meets the criteria; 0: does not meet the criteria.

## Data Availability

Not applicable.
